# Supplementary material for: Greater increases in intratumoral apparent diffusion coefficients after chemoradiotherapy predict better overall survival of patients with cervical cancer
Source: PLoS One. 2023 May 11;18(5):e0285786. doi: 10.1371/journal.pone.0285786 (PMC10174495; doi:10.1371/journal.pone.0285786)
Supplement: S1 Table — (DOCX) [file pone.0285786.s001.docx]

**S1 Table.** Overview of the imaging acquisition protocols of the diffusion-weighted imaging sequences

| **Scanner** | **Coil** | **Parameters** | **Pretreatment** | **Post-ERBT** | **IGBT** | **Post-treatment** |
| --- | --- | --- | --- | --- | --- | --- |
| University Hospital  protocol 1  Siemens Avanto Fit 1.5 T | Body | Number of patients | - | 1 | - | 1 |
|  |  | Slice thickness (mm) | - | 6 | - | 6 |
|  |  | Repetition time (ms) | - | 1700 | - | 1700 |
|  |  | Echo time (ms) | - | 62 | - | 62 |
|  |  | FoV (mm) | - | 420 | - | 420 |
|  |  | Rows | - | 300 | - | 300 |
|  |  | Columns | - | 384 | - | 384 |
|  |  | *b*-values | - | 50, 400, 800, 1200 | - | 50, 400, 800, 1200 |
| University Hospital  protocol 2  Siemens Avanto Fit 1.5 T | Body | Number of patients | 5 | 2 | 2 | 10 |
|  |  | Slice thickness (mm) | 6 | 6 | 6 | 6 |
|  |  | Repetition time (ms) | 1700 | 1700 | 1700 | 1700 |
|  |  | Echo time (ms) | 62‒72 | 62 | 62 | 62 |
|  |  | FoV (mm) | 380‒420 | 420 | 420 | 420 |
|  |  | Rows | 144‒300 | 300 | 300 | 300 |
|  |  | Columns | 192‒384 | 384 | 384 | 384 |
|  |  | *b*-values | 50, 400, 800 | 50, 400, 800 | 0, 50, 400, 800 | 50, 200, 400, 800, 1200, 1400 |
| University Hospital protocol 3  Siemens Avanto Fit 1.5 T | Body | Number of patients | 4 | 9 | 6 | 2 |
|  |  | Slice thickness (mm) | 3‒6 | 3‒6 | 6 | 6 |
|  |  | Repetition time (ms) | 1700 | 1700 | 1700 | 1700 |
|  |  | Echo time (ms) | 62 | 62‒72 | 62 | 62 |
|  |  | FoV (mm) | 420 | 380‒420 | 420 | 420 |
|  |  | Rows | 300 | 144‒300 | 300 | 300 |
|  |  | Columns | 384 | 192‒384 | 384 | 384 |
|  |  | *b*-values | 50, 400, 800, 1200, 1400 | 50, 200, 400, 800, 1200 | 50, 400, 800, 1000 | 50, 200, 400, 800 |
| University Hospital  Protocol 4  Siemens Aera 1.5 T | Body | Number of patients | - | - | - | 2 |
|  |  | Slice thickness (mm) | - | - | - | 6 |
|  |  | Repetition time (ms) | - | - | - | 1700‒4800 |
|  |  | Echo time (ms) | - | - | - | 60‒62 |
|  |  | FoV (mm) | - | - | - | 420‒450 |
|  |  | Rows | - | - | - | 300‒312 |
|  |  | Columns | - | - | - | 384 |
|  |  | *b*-values | - | - | - | 50, 200, 400, 800, 1200 |
| University Hospital  GE Signa Artist 1.5 T | Body | Number of patients | - | 3 | 10 | - |
|  |  | Slice thickness (mm) | - | 4 | 4 | - |
|  |  | Repetition time (ms) | - | 4607‒4690 | 3729‒8729 | - |
|  |  | Echo time (ms) | - | 62.1‒63.2 | 62.1‒66.1 | - |
|  |  | FoV (mm) | - | 300 | 300 | - |
|  |  | Rows | - | 256 | 256 | - |
|  |  | Columns | - | 256 | 256 | - |
|  |  | *b*-values | - | 50, 400, 800, 1200, 1400 | 0, 50, 200, 400, 800, 1000, 1200, 1400 | - |
| Central Hospital  GE Discovery MR450 1.5 T | Cardiac | Number of patients | 2 | 2 | - | 4 |
|  |  | Slice thickness (mm) | 4 | 4 | - | 4 |
|  |  | Repetition time (ms) | 3000 | 3000‒4225 | - | 2400‒3000 |
|  |  | Echo time (ms) | 58.4‒62.1 | 61.1‒70.2 | - | 52.1‒61.9 |
|  |  | FoV (mm) | 250‒280 | 280‒380 | - | 250‒360 |
|  |  | Rows | 256 | 256 | - | 256 |
|  |  | Columns | 256 | 256 | - | 256 |
|  |  | *b*-values | 0, 500, 800, 1000 | 0, 50, 400, 800, 1000, 1200 | - | 0, 50, 400, 800, 1000, 1200 |
| Central Hospital  Siemens Symphony Tim 1.5 T | Body | Number of patients | 4 | 4 | - | 2 |
|  |  | Slice thickness (mm) | 4‒5 | 4‒5 | - | 5 |
|  |  | Repetition time (ms) | 3700‒6103 | 3500‒6103 | - | 5500‒6103 |
|  |  | Echo time (ms) | 88 | 88‒122 | - | 88 |
|  |  | FoV (mm) | 220‒320 | 320‒400 | - | 320 |
|  |  | Rows | 102 | 102‒128 | - | 102 |
|  |  | Columns | 102 | 102‒128 | - | 102 |
|  |  | *b*-values | 0, 400, 500, 800, 1000, 1200 | 0, 50, 500, 800, 1000, 1200 | - | 0, 50, 800, 1200 |
| Central Hospital  Siemens Magnetom Sola 1.5 T | Spine_32_RS | Number of patients | 1 | 1 | - | 1 |
|  |  | Slice thickness mm | 3 | 4 | - | 6 |
|  |  | Repetition time (ms) | 4500 | 1599 | - | 6000 |
|  |  | Echo time (ms) | 74 | 61.1 | - | 94.4 |
|  |  | FoV (mm) | 220 | 377 | - | 400 |
|  |  | Rows | 256 | 224 | - | 256 |
|  |  | Columns | 256 | 224 | - | 256 |
|  |  | *b*-values | 50, 800, 1200 | 50, 800, 1200 | - | 50, 800, 1200 |
| Central Hospital  Siemens Avanto 1.5 T | Body | Number of patients | 5 | 1 | - | 1 |
|  |  | Slice thickness (mm) | 6 | 6 | - | 6 |
|  |  | Repetition time (ms) | 1700‒4900 | 4900 | - | 4900 |
|  |  | Echo time (ms) | 62‒72 | 72 | - | 72 |
|  |  | FoV(mm) | 380‒400 | 390 | - | 380 |
|  |  | Rows | 144‒300 | 144 | - | 144 |
|  |  | Columns | 192‒384 | 192 | - | 192 |
|  |  | *b*-values | 50, 200, 400, 800, 1200 | 50, 200, 400, 800, 1200 | - | 50, 200, 400, 800, 1200 |
| Central Hospital  Siemens  Avanto 1.5 T | Body | Number of patients | 1 | - | - | - |
|  |  | Slice thickness mm | 6 | - | - | - |
|  |  | Repetition time (ms) | 3900 | - | - | - |
|  |  | Echo time (ms) | 76 | - | - | - |
|  |  | FoV (mm) | 380 | - | - | - |
|  |  | Rows | 192 | - | - | - |
|  |  | Columns | 192 | - | - | - |
|  |  | *b*-values | 50, 800 | - | - | - |

EBRT = external beam radiotherapy; IGBT = image-guided brachytherapy; FoV = field of view
