# Supplementary material for: Greater increases in intratumoral apparent diffusion coefficients after chemoradiotherapy predict better overall survival of patients with cervical cancer
Source: PLoS One. 2023 May 11;18(5):e0285786. doi: 10.1371/journal.pone.0285786 (PMC10174495; doi:10.1371/journal.pone.0285786)
Supplement: S2 Table — (DOCX) [file pone.0285786.s002.docx]

**S2 Table**. Overall survival, univariate and multivariate analysis. Predictive value of ∆ ADC measurements (indicating the difference of the Apparent Diffusion Coefficient values between the pretreatment and post external beam radiotherapy (EBRT) and concurrent chemotherapy scans) and clinical variables.

Univariate analyses:

| **Variable** | **Classification** | **Univariate analysis** |
| --- | --- | --- |
|  |  | ***P*** |
| ∆ ADC L-ROI_mean_ | </≥ 237 mm^2^/s | 0.446 |
| ∆ ADC S-ROI_min_ | </≥ 252 mm^2^/s | 0.018 |
| Stage | I-IV | 0.830 |
| Tumor size | < 4 cm/ ≥ 4 cm | 0.067 |
| Lymph nodes | Positive/Negative | 0.239 |
| Parametria invasion | Yes/No | 0.177 |
| Adjuvant therapy | Yes/No | 0.295 |
| Residual tumor | Yes/No | 0.257 |

Cox regression multivariate analyses: Predictive value of ∆ ADC (difference of ADC between pretreatment and post-EBRT values measured by L-ROI_mean_) and clinical variable for overall survival.

| **Variable** | **Multivariate analysis** | | |
| --- | --- | --- | --- |
|  | **Hazard ratio** **95 % CI** ***P*** | | |
| ∆ ADC L-ROI_mean_ | 0.808 | 0.20–3.35 | 0.769 |
| Stage | 3.219 | 0.35–29.98 | 0.304 |
| Tumor size | 0.546 | 0.05–5.92 | 0.619 |
| Lymph nodes | 0.520 | 0.12–2.30 | 0.390 |
| Parametria invasion | 0.000 | 0.00-7.06E+14 | 0.952 |
| Adjuvant therapy | 0.898 | 0.21–3.92 | 0.886 |
| Residual tumor | 0.628 | 0.17–2.38 | 0.493 |

Cox regression multivariate analyses: Predictive value of ∆ ADC (difference of ADC between pretreatment and post-EBRT values measured by S-ROI_min_) and clinical variables for overall survival.

| **Variable** | **Multivariate analysis** | | |
| --- | --- | --- | --- |
|  | **Hazard ratio** **95 % CI** ***P*** | | |
| ∆ ADC S-ROI_min_ | 0.199 | 0.04–1.09 | 0.063 |
| Stage | 1.751 | 0.39–7.83 | 0.463 |
| Tumor size | 0.873 | 0.90–8.44 | 0.907 |
| Lymph nodes | 0.422 | 0.09–1.99 | 0.288 |
| Parametria invasion | 0.234 | 0.03–2.18 | 0.202 |
| Adjuvant therapy | 1.598 | 0.31–8.26 | 0.576 |
| Residual tumor | 0.472 | 0.11–2.05 | 0.316 |

∆ ADC = calculated difference between pretreatment and post-EBRT ADC values, ROC curve was used to analyze the Youden index, that was used for cutoff value determination

L-ROI = large region of interest, S-ROI = small region of interest, CI = confidence interval

Residual tumor = visible tumor at 3 months post-treatment MRI
