# Supplementary material for: Greater increases in intratumoral apparent diffusion coefficients after chemoradiotherapy predict better overall survival of patients with cervical cancer
Source: PLoS One. 2023 May 11;18(5):e0285786. doi: 10.1371/journal.pone.0285786 (PMC10174495; doi:10.1371/journal.pone.0285786)
Supplement: S3 Table — (DOCX) [file pone.0285786.s003.docx]

**S3 Table.** Recurrence free survival at 3 year timepoint, univariate and multivariate analysis. Predictive value of ∆ ADC measurements (indicating the difference of the Apparent Diffusion Coefficient values between the pretreatment and post external beam radiotherapy (EBRT) and concurrent chemotherapy scans) and clinical variables.

Univariate analyses:

| **Variable** | **Classification** | **Univariate analysis** |
| --- | --- | --- |
|  |  | ***P*** |
| ∆ ADC L-ROI_min_ | </≥ 275 mm^2^/s | 0.012 |
| Pretreatment  ADC S-ROI_min_ | </≥ 576 mm^2^/s | 0.049 |
| Stage | I-IV | 0.935 |
| Tumor size | < 4 cm/ ≥ 4 cm | 0.095 |
| Lymph nodes | Positive/Negative | 0.412 |
| Parametria invasion | Yes/No | 0.606 |
| Adjuvant therapy | Yes/No | 0.988 |

Cox regression multivariate analyses: Predictive value of ∆ ADC (difference of ADC values between pretreatment and post-EBRT values measured by L-ROI_min_) and clinical variables for recurrence free survival in 3-year follow-up.

| **Variable** | **Multivariate analysis** | | |
| --- | --- | --- | --- |
|  | **Hazard ratio** **95 % CI** ***P*** | | |
| ∆ ADC L-ROI_min_ | 0.204 | 0.05–0.88 | 0.033 |
| Stage | 3.467 | 0.39–30.69 | 0.264 |
| Tumor size | 0.446 | 0.04–4.60 | 0.497 |
| Lymph nodes | 0.455 | 0.12–1.70 | 0.238 |
| Parametria invasion | 0.459 | 0.05–4.43 | 0.501 |
| Adjuvant therapy | 2.592 | 0.50–13.60 | 0.260 |

Cox regression multivariate analyses: Predictive value of pretreatment ADC values measured by S-ROI_min_ and clinical variables for recurrence free survival in 3-year follow-up.

| **Variable** | **Multivariate analysis** | | |
| --- | --- | --- | --- |
|  | **Hazard ratio** **95 % CI** ***P*** | | |
| ADC S-ROI_min_ | 0.340 | 0.12–1.00 | 0.050 |
| Stage | 1.436 | 0.27–7.52 | 0.668 |
| Tumor size | 0.374 | 0.08–1.86 | 0.230 |
| Lymph nodes | 0.557 | 0.19–1.66 | 0.294 |
| Parametria invasion | 0.795 | 0.14–4.52 | 0.796 |
| Adjuvant therapy | 2.005 | 0.40–9.99 | 0.396 |

∆ ADC = calculated difference between pretreatment and post-EBRT ADC value, ROC curve was used to analyze the Youden index, that was used for cutoff value determination

L-ROI = large region of interest, S-ROI = small region of interest, CI = confidence interval
