# Supplementary material for: Greater increases in intratumoral apparent diffusion coefficients after chemoradiotherapy predict better overall survival of patients with cervical cancer
Source: PLoS One. 2023 May 11;18(5):e0285786. doi: 10.1371/journal.pone.0285786 (PMC10174495; doi:10.1371/journal.pone.0285786)

**S1 Figure.** Univariate analysis of cumulative overall survival according to the different prognostic factors for cervical cancer.

A: FIGO stage I-IV (*P* = 0.830): blue line indicates stage I, red stage II, green stage III, and orange stage IV.

B: Tumor size (*P* = 0.067): blue line indicates tumor size < 4 cm and red line ≥ 4 cm.

C: Lymph nodes (*P* = 0.239): blue line indicates negative lymph nodes and red line positive lymph nodes.

D: Parametria invasion (*P* = 0.177): blue line indicates parametria invasion, red line no parametria invasion.

E: Adjuvant therapy (*P* = 0.295): blue line indicates that patient received adjuvant therapy, red line indicates no adjuvant therapy.

F: Residual tumor (*P* = 0.257): blue line indicates no residual tumor at 3 months post-treatment MRI and red line indicates residual tumor at 3 month post-treatment MRI.


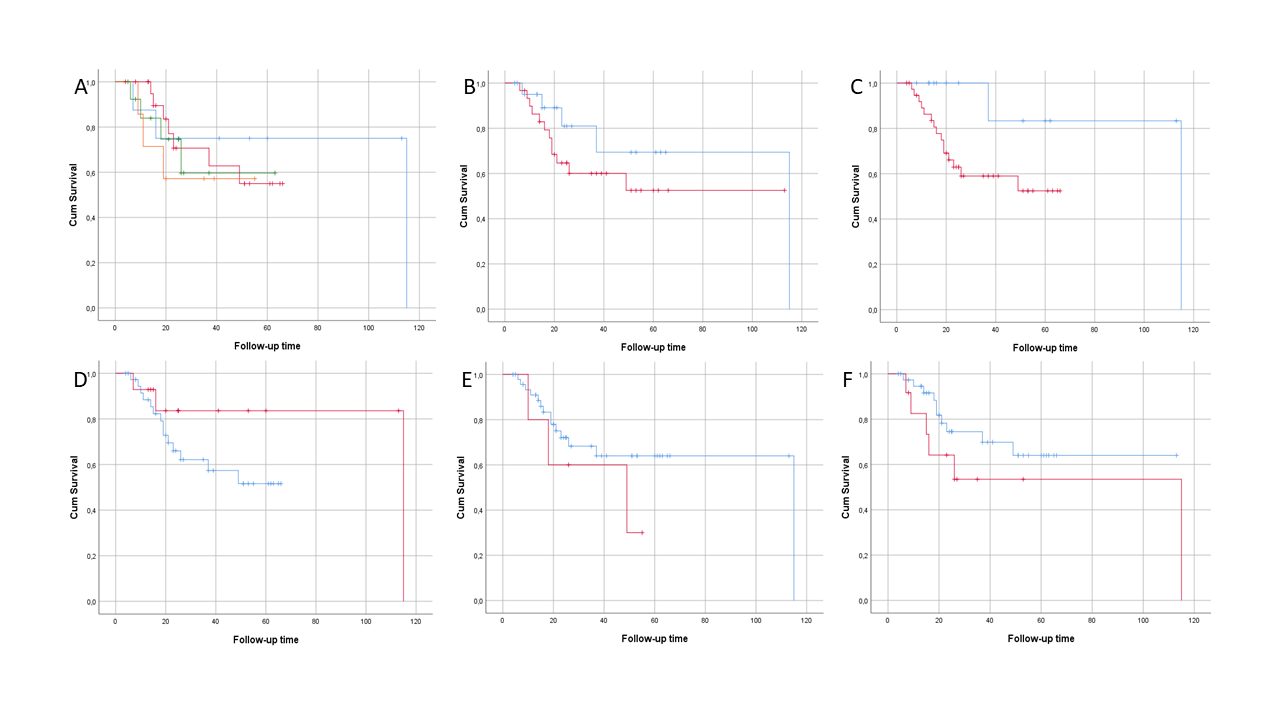

Supplement: S1 Fig — (DOCX) [file pone.0285786.s004.docx]
