# Supplementary material for: Greater increases in intratumoral apparent diffusion coefficients after chemoradiotherapy predict better overall survival of patients with cervical cancer
Source: PLoS One. 2023 May 11;18(5):e0285786. doi: 10.1371/journal.pone.0285786 (PMC10174495; doi:10.1371/journal.pone.0285786)
Supplement: Attachment — Submitted filename: Response to Reviewers.docx [file pone.0285786.s005.docx]

POINT-BY-POINT RESPONSE

Reviewer #1

Major concern:
The focus of your research is excellent. There are no standardized methods for measuring ADCs in patients with cervical cancer, this study indicated the efficacy of monitoring treatment response after chemoradiotherapy if the pre-treatment ADC values are compared to post-treatment values.
However, this is only a univariate analysis, and other confounding factors have not been evaluated, and factors such as tumor size have not been removed. At the very least, a multivariate analysis is necessary, and I urge you to reconsider your analysis method.

*Thank you for your kind comment and suggestions to improve the manuscript. We have now performed multivariate analysis and the results are shown in the revised version.*

Materials and Methods:
{94 patients were excluded because MRI with DWI was not available at all these time points. Thus, 23 consecutive patients who underwent MRI with DWI at the six consecutive time points were included in the study.}

The lack of MRIs at all 6 time points has resulted in the exclusion of 94 patients and a much lower number of N. Even without MRIs at all 6 time points, is there any way to compare more than 100 patients?
I feel that the drastic decrease in the number of Ns is a bit of a waste in this study.
There seems to be a lot of exclusions due to missing MRI, especially during intra-tissue irradiation. Is there any way to avoid reducing the number of N or subgroup analysis?

*Thank you for these important questions. We agree that the number of patients included in the study was low. It was very disappointing to see the lack of MRI scans at all the six timepoints. Our institution is a tertiary care center and patients can come from all over the country to receive IGBT treatments to our center. Not all of them have diffusion weighted images in their pretreatment or post external beam radiotherapy (EBRT) MRI protocols.*

*Actions taken: Originally, we included consecutive patients with histopathologically proven cervical cancer who were treated at our institution between 2009 and 2020 and required that they had finished their treatment protocol before the end of 2020, to obtain a reasonable follow-up period. In the revised manuscript, we also accepted patients who were diagnosed before the end of 2020 but had not finished their treatment protocol then, as a reasonable follow-up period could now be obtained also for them. Furthermore, we now included all patients who had an MRI scan with diffusion weighted imaging pretreatment and post-EBRT and chemotherapy. Those scans were used in reproducibility analyses and in the univariate and multivariate assessments of prognostic factors. That way we managed to increase the number of patients to 52. Five of those new patients had MRI with DWI at six timepoints and they were included also in the subgroup analysis, increasing the number to 28. Unfortunately, we were not able to reach the 100-patient goal.*

Result:
How about adopting FIGO 2018 for the staging classification of stage I B? (ⅠB1-2→1-3)

*Thank you for the comment.*

*Actions taken: The stage has been changed according to FIGO 2018 classification. See manuscript page 4, line 96 and pages 9-10, Table 1.*

The statement "ADC remains significantly higher than pre-treatment values during the IGBT period."
However, after IGBT2, both L-ROImean and S-ROImean ADC have decreased and lost significant difference with respect to L-ROI. The S-ROI also barely shows a significant difference. IGBT3 also shows a significant re-elevation in both cases.
In this regard, why does the ADC temporarily decrease after IGBT2?
Can this phenomenon be a limitation in using ADC as a prognostic factor?

*Thank you for the important comment and questions. IGBT is very effective therapy, and often the tumors shrink or even disappear during the treatment. When the tumors get smaller and shrink, they also get denser and this could explain why ADC values decrease* *at that point, reflecting the restriction of the movement of water molecules. In addition, several of the tumors were so small in the IGBT and post-treatment scans that the ADC measurements could not be performed. In the subgroup analysis, only 14 tumors could be measured at IGBT, compared to 25 tumors measured post-EBRT, possibly leaving the more aggressive ones still visible. The phenomenon that ADC values “fluctuate” during IGBT, can be a limitation in using ADC as a prognostic factor, but this can be avoided if the change in ADC values is measured already after post-EBRT.*

*Actions taken: The following sentences have been added to the discussion, page 17, lines 335-340:*

*Interestingly, the ADC values were somewhat lower after IGBT2 compared to those post-EBRT. Several of the tumors were so small in the IGBT scans that the ADC measurements could not be performed. In the subgroup analysis, only 14 tumors could be measured at IGBT compared to the 25 tumors measured post-EBRT, possibly leaving the most aggressive ones still visible. In addition, the tumors often shrink during IGBT, and the remaining smaller and denser tumors may in part explain the lower ADC values*.

Also, you state in the text, "ADC at 3 months post-treatment was not significantly different from that after EBRT."
Why is there no significant difference in ADC when 75% of the patients with residual tumor have disappeared at 3 months after treatment? It seems to me that the shrinkage of the tumor does not correlate with the increase in ADC.

*Thank you for the* *question. As mentioned above, IGBT is very effective therapy and most of the tumors disappeared during the treatment. In the tumors remaining at 3 moth scans (n = 10) the ADC values showed a significant increase compared to the pretreatment scans which could indicate some cellular response to treatment, while the tumor had somewhat shrunken. There was, however, no difference compared to the post-EBRT scans. Unfortunately, we are not able to fully explain the reason for this phenomenon.*

*Actions taken: The following sentences have been added to the revised manuscript, page 10, lines 215-219:*

*In patients with residual tumor after IGBT, the ADCs at 3 moths’ post-treatment MRI were significantly higher than pretreatment ADCs* (*L-ROI_min_ 843 mm^2^/s, P = 0.002, S-ROI_mean_ 1001 mms^2^/s, P = 0.008, S-ROI_min_ 884 mm^2^/s, P = 0.006***)** *but there were no significant changes in the ADCs at 3 months post-treatment compared with post-EBRT.*

{Smaller tumor size (<4 cm) was associated with lower pretreatment ADCs and the changes in ADCs after EBRT were greater for smaller tumors.}
In considering changes in ADC as a prognostic factor, did you exclude the effect of tumor size? It would make sense if the complete response rate were higher with a higher change in ADC regardless of tumor size.

*Thank you for your valuable comment. We have repeated the univariate calculations now with the larger (n = 52) cohort and some of the results changed. More advanced tumor stage tended to be associated with higher ADCs, larger tumors of over 4 cm had lower ADC values post-EBRT, and tumors that recurred during follow-up had higher ADC values in pretreatment MRI.*

*Actions taken: Table 3 is now renewed with the larger cohort, pages 13-14. Some new prognostic factors have been added (lymph nodes, parametria invasion and adjuvant therapy).The following sentences are included in the revised manuscript, page 12, lines 257-265:*

*Tumor grade or histology were not associated with the pretreatment ADCs. More advanced tumor stage tended to be associated* (*P = 0.063*) *with higher ADCs in L-ROI_mean_. Larger tumors of over 4 cm had lower ADC values post EBRT in L-ROI_mean_* (*P = 0.034*) *and tended to be lower in S-ROI_mean_* (*P = 0.076*)*. Tumors that recurred in follow up showed higher ADC values in pretreatment MRI* (*S-ROI_min_, P = 0.045; L-ROI_min_, P = 0.055*)*. In patients, who had adjuvant therapy* (*n = 5*) *the increase in ADC values between pre- and post-EBRT did not reach statistical significance using two of the measurement methods* (*L-ROI_mean_, P = 0.084; S-ROI_min,_ P = 0.105*)*, while in patients with no adjuvant therapy* (*n = 47*) *the increase was highly significant with all ROI measurement methods* (*P = 0.001*) (*Table 3*)*.*

{An increase in the ADC of >32% between the pre- and post-EBRT scans for L-ROImean predicted better overall survival (P = 0.040). Other ADC values in different time points did not associate with overall or recurrence free survival.}
How can you say that ADC change is a prognostic factor with results that are significant only after EBRT among CCRT and no significant difference in the remaining treatments? This conclusion makes me feel that treatment after EBRT is not effective.
Besides, this is only a univariate analysis, and other confounding factors have not been evaluated, and factors such as tumor size have not been removed. At the very least, a multivariate analysis is necessary, and I urge you to reconsider your analysis method.

*Thank you for the valuable comments and questions. Our cohort of patients received both concurrent chemoradiotherapy and four cycles of image-guided brachytherapy. After including more patients to the predictive analyses, the increase in ADC values after EBRT remained as a significant prognostic factor. However, this does not mean that the treatment after EBRT is not effective. Rather, this indicates that those patients, whose tumors show an increase in ADC already after EBRT, will benefit most, when they receive also subsequent IGBT.*

*Actions taken: We have revised the results with this new larger cohort. The following sentences have been added to page 15, lines 283-842: An increase in the intratumoral ADC > 47 % between pre- and post-EBRT scans for S-ROImin predicted better overall survival (P = 0.018) in Kaplan Meier analyses.*

*We calculated the overall and recurrence free survival analysis using also 1-year, 2-year, and 3-year timepoints. To evaluate other confounding factors, we now performed Cox regression multivariate analysis where we included ADC values and their change together with known prognostic factors for cervical cancer. The following sentences have been added to results section, page 15, lines 290-296.*

*We included ADC values and their change, measured with different ROI delineation methos, together with known prognostic factors of cervical cancer (stage, tumor size, lymph node, parametria invasion and adjuvant therapy) to Cox regression multivariate analysis. For overall survival, at 1-year timepoint pretreatment ADC value remained significant in multivariate analysis (L-ROI_mean_, P = 0.006, HR 61.419 (95% CI 3.19-1181.06). ADC value increase measured with S-ROI_min_ tended to be significant also in multivariate analysis for the whole follow-up time (P = 0.068, HR 0.217 (95% CI 0.04-1.12) and for the 2-year timepoint (P = 0.067, HR 0.138 (95% CI 0.02-1.15).*

Discussion:
{Nakamura et al. [24] reported that the mean ADCs of the primary tumor were significantly associations with FIGO stage, tumor size, stromal and parametrial invasion, lymph node metastasis, lymph vascular space involvement, and adjuvant therapy.}
The opposite results of the present study indicate that the influence of these confounding factors should be eliminated by changing the method of analysis.

*Thank you for the comment. We have now reanalyzed our results with known prognostic factors for cervical cancer. Furthermore, the confounding factors are now included in the Cox regression multivariate analysis where we included ADC values and their change, together with known prognostic factors for cervical cancer (see response to the previous point).*

*Actions taken: Following parameters have been added to Table 3: lymph nodes, parametria invasion and adjuvant therapy. Unfortunately, the lymph vascular space involvement was not available in patient records. The following sentences are now included in the Results section, page 12, lines 257-265:*

*Tumor grade or histology were not associated with the pretreatment ADCs. More advanced tumor stage tended to be associated (P = 0.063) with higher ADCs in large ROI_mean_. Larger tumors of over 4 cm had lower ADC values post EBRT in L-ROI_mean_ (P = 0.034) and tended to be lower in S-ROI_mean_ (P = 0.076). Tumors that recurred in follow up showed higher ADC values in pretreatment MRI (S-ROI_min_; P = 0.045; L-ROI_min_, P = 0.055). In patients, who had adjuvant therapy (n = 5) the increase in ADC values between pre- and post-EBRT did not reach statistical significance using two of the measurement methods (L-ROI_mean_, P = 0.084; S-ROI_min_, P = 0.105), while in patients with no adjuvant therapy (n = 47) the increase was highly significant with all ROI measurement methods (P = 0.001) (Table 3).*

*The following sentences have been added to the discussion, page 18, lines 349-353: In our cohort, tumor grade, size, histology, parametria invasion or lymph node metastasis were not associated with the pretreatment ADCs while a more advanced tumor stage tended to be associated with ADCs. The somewhat different results are possibly due to the inclusion of different patient populations, variations in ADC measurement techniques, and the small study population.*

{Our results suggest that the predictive information of ADC measurements is greater when the pretreatment and post-EBRT ADCs are compared.}
What is the intent of this sentence? Please provide details.

*Thank you for the valuable comment.* *We have repeated the measurements and analysis with a larger number of patients and performed Cox-regression multivariate analysis. The predictive value of ADC difference between pre- and post-EBRT was shown to be statistically significant in 2-year and 3-year timepoints of survival data. A higher pretreatment ADC value predicted better overall survival at 1-year timepoint, but not at 2-year or 3-year timepoints. The post-EBRT ADC-values alone were not significant predictors of survival.*

*Actions taken: The results are shown in revised manuscript, page 10, lines 207-219 and page 15 lines 283-289 and figure 6.*

*An increase in the intratumoral ADC of >47% between the pre- and post-EBRT scans for S-ROI_min_ predicted better overall survival (P = 0.018) in Kaplan Meier analyses (Fig. 6). Because of the large variety of follow-up times, we calculated the survival analysis also using 1-year, 2-year, and 3-year timepoints. A higher pretreatment ADC value predicted better overall survival at 1-year timepoint (L-ROI_mean_, P = 0.05). A greater increase of over 47% in the ADC values between pre-and post-EBRT scans predicted better overall survival at 2-year follow-up period (L-ROI_mean_, P = 0.045; S-ROI_min_, P = 0.008) and at 3-year follow-up period (S-ROI min, P = 0.024).*

*The following sentence has been added to the discussion page 18, lines 363-365: Our results suggest that the predictive information of ADC measurements is greater when the pretreatment and post-EBRT ADCs are compared, instead of using the ADC values at a single time-point.*


{In the Kaplan‒Meier univariate analysis of cumulative overall survival, a greater increase (>32%) in the ADC between pre- and post-EBRT was associated with better overall survival (P = 0.040) when using L-ROImean.}
This is only a univariate analysis, and other confounding factors have not been evaluated, and factors such as tumor size have not been removed. At the very least, a multivariate analysis is necessary, and I urge you to reconsider your analysis method.
The evidence showed the other pretreatment, post-ERBT, and post-treatment ADC parameters were not associated with overall or recurrence-free survival.

*Thank you for the valuable comment. We have now performed Cox regression multivariate analysis with the larger cohort. We also calculated the survival using different timepoints (1-year, 2-year and 3 year).*

*Actions taken: The following sentences have been added in the results section, page 15, lines 290-297:*

*We included ADC values and their change, measured with different ROI delineation methos, together with known prognostic factors of cervical cancer (stage, tumor size, lymph node, parametria invasion and adjuvant therapy) to Cox regression multivariate analysis. For overall survival, at 1-year timepoint pretreatment ADC value remained significant in multivariate analysis (L-ROI_mean_, P = 0.006, HR 61.419 (95% CI 3.19-1181.06). ADC value increase measured with S-ROI_min_ tended to be significant also in multivariate analysis for the whole follow-up time (P = 0.068, HR 0.217 (95% CI 0.04-1.12) and for the 2-year timepoint (P = 0.067, HR 138 (95% CI 0.02-1.15). None of the other prognostic factors remained statistically significant in multivariate analysis in our cohort.*

{Our study had several limitations. First, the study population was small.}
The lack of MRIs at all 6 time points has resulted in the exclusion of 94 patients and a much lower number of N. Even without MRIs at all 6 time points, is there any way to compare more than 100 patients?

*Thank you for this important comment.*

*Actions taken: We included more patients in the study by including patients diagnosed with cervical cancer by the end of 2020, even if their treatment was undertaken in 2021. We also included now all patients who had pretreatment and post EBRT MRIs with DWI available. We were able to increase the total number of patients to 52. Altogether 28 of these patients had MRIs with DWI available at all six timepoints, so we included them in the subgroup analysis.*

Conclusion:
{Importantly, the greater increase in ADCs measured using L-ROIs provided an additional marker for predicting better overall survival.}
The results of this study, statistically, do not suggest this above content as a conclusion.
It is too early to conclude that the greater increase in ADCs is a marker for predicting better overall survival by your analysis method.

*Thank you for the comment. It is true, that our cohort is still small, and the results need confirmation in larger studies. We have also added multivariate analysis now.*

*Actions taken: We have now modified the conclusion section as follows, page 20, lines 403-411:*

*In cervical cancer, ADC values differ significantly depending on the method used to delineate the ROIs, with small ROIs on most restricted areas producing significantly lower ADC values compared to the large whole tumor covering ROIs. Intratumoral ADCs increase significantly between the pretreatment, post-EBRT, and IGBT MRIs regardless of the ROI delineation method. In clinical practice our results support measuring and comparing the ADC values before treatment and after EBRT, rather than using the ADC values at a single time-point. Importantly, in our cohort, the greater increase of ADCs after EBRT and measured using small standard-size ROIs seems to provide a surrogate marker for better recurrence free survival and overall survival. Standardized methods for timing and delineation of ADC measurements are recommended in future studies.*

*In the Abstract, the conclusion has been also modified, page 2, lines 42-44:*

*ADC values significantly increase during cervical cancer treatment. Greater increases in ADCs after EBRT predicted better overall survival using S-ROIs. Standardized methods for timing and delineation of ADC measurements are advocated in future studies.*

Reviewer #2

Some sentences contain expressions that are little difficult to understand, please fix if possible.
(I can understand what you mean.)
line 26-27 And is this change…

*Thank you for the comment. We have now tried to rephrase our text.*

*Actions taken: The following sentences have been added to the abstract, page 2, line 25-27: To evaluate whether 1) the intratumoral apparent diffusion coefficient (ADC) values changes during cervical cancer treatment and 2) are pretreatment ADC values or their change after treatment is predictive for the treatment outcome or overall survival of patients with cervical cancer.*

line 251-253 On average,the…, ; There is a description in the previous sentence, but I think it is easier to understand if you describe the measurement tome again.

*Thank you for the comment.*

*Actions taken:* *The following sentence is added to* *page 10, lines 210-212:* *Between pretreatment and post-EBRT MRI, the mean ADCs increased by 26 % for L-ROI_mean_ (P < 0.001), by 41 % for S-ROI_mean_ (P < 0.001), by 55 % for L-ROI_min_ (P < 0.001) and by 47 % for S-ROI_min_ (P < 0.001).*

Line 367-368 Our results suggest that….

*Thank you for the observation. We have modified the sentence as follows, page 18, lines 363-365:*

*Our results suggest that the predictive information of ADC measurements is greater when the pretreatment and post-EBRT ADCs are compared, instead of using the ADC values at a single time-point.*

line 96, you said “patients with more advanced disease (FIGO stage ⅠB2 or grater) are treated with chemoradiotherapy. But in the clinical scenes, even if the disease is not advanced, surgery may not be performed due to comorbidities, age, and other reasons of the patients. It is better to change the expression because it may cause misunderstanding (Please refer to the cervical cancer treatment guidelines etc.).

*Thank you for your remark. We agree with you on this. The other factors like contraindication to surgery and anesthesia should be considered.*

*Actions taken: We have reviewed FIGO Cancer Report and corrected the following sentence: page 3, lines 65-69 and reference number10:*

*Patients with early-stage disease, a tumor confined to the uterus, or tumors <4 cm in size (FIGO stages IB1, IB2 and IIA1) are usually treated with primary surgical resection and lymphadenectomy, but in cases with contraindications for surgery or anesthesia, chemoradiotherapy is an equally good alternative. Patients with more advanced disease (FIGO stage IB3 or greater) are treated with chemoradiotherapy [4,5,6,10].*

line 132,133; You wrote “Ninety-four patients”, “23 consecutive patients”
Is there any reason why you changed the numbering method? You should unify numbering method i.e. ;94 patients 23 consecutive patients, Ninety-four patients twenty-three consecutive patients

*Thank you for pointing that out. In the beginning of a sentence, we have written the numbers by letters, while inside the sentence by numbers.*

*Actions taken: We have now modified the inclusion criteria. the revised text is written as follows, page 4-5, lines 95--105:*

*Altogether 122 patients staged by pelvic MRI and body CT/PET-CT who were unsuitable for surgical treatment according to the FIGO 2018 staging system received concurrent chemoradiotherapy (CCRT) comprising external-beam radiotherapy (EBRT) (total dose: 45 Gy) with concurrent cisplatin-based chemotherapy and four cycles of image-guided brachytherapy (IGBT) according to the European Society of Gynecological Oncology (ESGO) guidelines [17] and were evaluated as candidates for the present study. Altogether 52 patients (mean age 56 years) had undergone diffusion weighted MRI examinations before treatment and post- EBRT and concurrent chemotherapy and were included in the study. A subgroup of those patients (n = 28, mean age 53 years) underwent consecutive diffusion weighted MRI examinations at six timepoints; 1) pretreatment, 2) post-EBRT and concurrent chemotherapy; 3-5) during IGBT, and 6) the follow-up scan 3 months after the whole treatment (Fig. 1).*

You wrote grade3 and grade2 in the title of captions Fig.2 and Fig.3. As the histopathological type of cervical cancer, we described as “adenocarcinoma”, “squamous cell carcinoma” without grade, commonly. Please explain specifically.

*Thank you for the comment.*

*Actions taken: We have removed the grade from captions and included the histopathological type instead, page 6, lines 135 and 144:*

*Fig. 2. MRI of a 55-year-old female with stage IIIc1 adenocarcinoma of the cervix.*

*Fig. 3. MRI of a 55-year-old female with stage IIIc1 adenocarcinoma of the cervix.*

You measured the ADC of L-ROI from axial images, is not it common to evaluate with sagittal images? If so, is there a reason for that?

*Thank you for Your question. In our protocol the DWI sequences are obtained only in axial plane and since the slice thickness is 5 mm, sagittal reformats cannot be obtained afterwards. Consequently, the ADC measurements are done in axial plane.*

In Fig.2, you put on only sagittal images during treatment and after treatment. Isn’t it better to put axial images during and after treatment?

*Thank you for the comment. We feel that sagittal spin echo images were more informative.*

*Actions taken: We have now included also axial images into the figure, page 6, figure 3.*

Table 1. Since the stage of cervical cancer has just been revised in FIGO2018, it would be better to state somewhere that the description of the stage follows FIGO2018. If possible, please describe the details about cancer recurrence (recurrent site), and deceased patients.

*Thank you for this relevant comment.*

*Actions taken: We have included the description FIGO 2018 in the manuscript, page 4, line 96 and Table 1, page 9.*

*We have also included available details about cancer recurrence sites. Since our institution is a tertiary care center, and patients can come from all over the country for IGBT treatment, we don’t have information about the deceased patient other than the date of death.*

*Action taken:* *The following sentences are added to page 8, lines 178-181. Twenty patients had cancer recurrence and 12 had progressive disease. The most common site for recurrence was lymph nodes following lungs and brain. During the follow up period from the time of diagnosis until end of year 2022, 17 patients had died.*

You wrote that there were no significant changes in the ADCs after IGBT. But in the clinical scenes, IGBT is very effective therapy. Do you have any considerations on no change in ADC after IGBT?

*Thank you for the important comment. As you stated, IGBT is very effective therapy, and in our cohort most of the tumors tend to disappear during the treatment cycle. Several of the tumors were so small in the IGBT scans that the ADC measurements could not be performed. During the IGBT, ADC values seemed to decrease little in the remaining tumors. We think this happened because the remaining tumors were the more aggressive ones and second, the tumor shrinks. As the treatment continues, ADC values remain relatively the same. If the ADC values during IGBT are compared to ADC values of a previous scan, there is no statistically significant difference but if the ADC values are compared to pre-treatment ADC values, the increase remains statistically significant.*

*Actions taken: The following sentences have been added to the discussion, page 17, lines 335-340:*

*Interestingly, the ADC values were somewhat lower after at IGBT2 compared to those post-EBRT. Several of the tumors were so small in the IGBT scans that the ADC measurements could not be performed. In the subgroup analysis, only 14 tumors could be measured at IGBT compared to the 25 tumors measured post-EBRT, possibly leaving the most aggressive ones still visible. In addition, the tumors often shrink during IGBT, and the remaining smaller and denser tumors may in part explain the lower ADC values.*

Fig.6
You wrote the ADC changes of stage1~2 was greater than that of progressive cancers. Isn’t it just that the survival rate is better because the red line contains more early stages? I want you to describe the stage of the cancers in the red line and blue line.

*Thank you for the comment*. *Since we included more patients to the study, we calculated the survival with increased number of patients and with multivariate analysis. We included ADC values and their change, measured with different ROI delineation methods, together with known prognostic factors of cervical cancer (stage, tumor size, lymph node, parametria invasion and adjuvant therapy) to Cox regression multivariate analysis, but none of the prognostic factors remained statistically significant, ADC value for S-ROI_min_ tended to be significant (P = 0.068, HR 0.217 (95% CI 0.04-1.12).*

*Actions taken: We updated the changes in the manuscript accordingly: pages 15-16, lines 290–305 and Fig. 6. In the Figure, the univariate analysis between two groups indicates patients with different levels of ADC increase. The Figure legend is now as follows, page 16, lines 307-311:*

*Fig. 6. Univariate analysis of cumulative overall survival according to the relative change in ADC values from pretreatment to post-EBRT MRI scan using intratumoral S-ROIs.*

*Blue line indicates group of patients with an increase of over 47% in ADC values (n = 14). Red line indicates group of patients with an increase of less than 47% in ADC values (n = 23) An increase in the ADC of over 47% for S-ROI predicted better overall survival (P = 0.018).*
