# Supplementary material for: Greater increases in intratumoral apparent diffusion coefficients after chemoradiotherapy predict better overall survival of patients with cervical cancer
Source: PLoS One. 2023 May 11;18(5):e0285786. doi: 10.1371/journal.pone.0285786 (PMC10174495; doi:10.1371/journal.pone.0285786)
Supplement: Attachment — Submitted filename: Response to reviewers.docx [file pone.0285786.s006.docx]

POINT BY POINT RESPONSE TO THE REVIEWER

Reviewer #1: Major point
Multivariate analysis has been added and good results have been obtained, however there are no Figures and Tables for multivariate analysis, please add them. It is difficult to convey this information in text only.
Since the results of the multivariate analysis are the most important in this study, I think it would be better to include all the results of the multivariate analysis in a Figure and Table, even if it is supplemental.
Please include not only the prognostic factors that showed significant differences, but also those that did not show significant differences.
Please include not only p-value and Hazard Ratio but also the actual values for the entire analysis. The curves from the univariate analysis alone are not convincing.

*Thank you for all the valuable comments and suggestions to improve the manuscript. We have added Supplementary tables and figures of the multivariate analyses for overall survival and recurrence free survival. The changes are shown in the revised version of our manuscript.*

*Action taken: See supplementary tables S2, S3 and supplementary figure S4.*


Minor point
{Using Cox regression multivariate analysis ADC value change measured with LROImin was statistically significant at 3-year timepoint (P = 0.033, HR 0.204 (95% CI 0.05-0.88) and pretreatment ADC value S-ROImin (P = 0.05, HR 0.340 (95%CI 0.12-1.00) at 3-year timepoint remained also significant.}
→In principle, do you recommend that both S-ROI and L-ROI be measured, with L-ROI as the percentage of ADC value change before and after treatment, and S-ROI as the pretreatment ADC value as a prognostic predictor at 3-year timepoint?
Please describe the recommended use as a prognostic factor.

*Thank you for the question. We used different methods in our measurements and assessed their prognostic value in several univariate and multivariate analyses at different timepoints. Although the L-ROI measurements provided significant or almost significant results in some analyses, the S-ROI measurements provided significant results in a larger number of analyses. ∆ ADC between pretreatment and post-EBRT ADC measurements proved to be a significant prognostic predictor in OS and RFS calculations. Based on those results we feel that the best method is to use the small ROI minimum value since that gave us best overall results.*

*For detailed results, we refer to the sentences in the manuscript, see page 15-16, lines 283-307.*

*Actions taken: The following sentence has been added to conclusion section of the manuscript, see page 20, lines 420-422:*

*Importantly, in our cohort, the greater increase in ADC values between pretreatment and post-EBRT values measured using small standard-size ROIs, seemed to provide a surrogate marker for better overall and recurrence free survival*.

In the Univariate analysis curve (Fig. 6), the number of N is 37 (14+23), why did 15 cases drop out of 52? Please describe the reason.

*Thank you for the comment*. *The 15 cases out of the whole group of 52 dropped out since the tumors had vanished already during the EBRT and there was no visible tumor in the post-EBRT scans for ADC measurement.*

*Actions taken: The following sentence in the manuscript has been modified, see page 8, lines 183-185: Following EBRT and concurrent cisplatin chemotherapy, 37 (71%) of the 52 patients had a measurable residual tumor on the post-EBRT MRI and 15 (29%) of the tumors had vanished during the chemoradiotherapy.*

After EBRT, ADC could be measured in only 14 of 28 cases due to tumor shrinkage.
I question whether the use of ADC value change immediately after EBRT as a prognostic factor after CCRT, even though the number of cases after EBRT has decreased by half.
Why not analyze only the 14 cases that could be measured ADC all 6 times?
Only 10 patients had residual tumor at 3 months, and in those 10 patients, there was no significant difference in ADC value from EBRT to IGBT.
Is the prognosis better for patients with a larger ADC change value in both residual tumor group and no residual tumor group?
Can you conclude that a larger ADC change rate between pre-treatment and post-EBRT is associated with a significantly higher survival rate regardless of tumor residuals?

*Thank you for the comment. We are sorry, that we had not been clear in our description of the results. We have measured the ADC values from* ***37*** *patients after EBRT. During chemoradiotherapy 15 tumors of the original 52 tumors had shrinked or vanished, and ADC was therefore unmeasurable after chemoradiotherapy. During the intracavitary treatment the healing continued and in the end of the whole treatment (at 3month scan) only 10 tumors out of 52 were still visible and measurable.*

*Action taken: The following sentences have been added to the chapter ‘ADCs and their changes during treatment’ see page10, lines 215-222:*

*To further analyze the importance of a residual tumor after treatments, the patients with a measurable tumor in the post-EBRT scans (n =37) were divided to those with a residual tumor in the 3-month post-treatment scan (n = 10) and those with no residual tumor in the 3-month post-treatment scan (n = 27). The ADCs were significantly higher at the post-EBRT measurements than in the pretreatment scans in both groups (L-ROI_mean_ P = 0.006, S-ROI_min_ P = 0.017 in residual tumor group and L-ROI_mean_ P < 0.001, S-ROI_min_ P < 0.001 in no residual tumor group respectively. The ADC change was also significant for L-ROI_min_ and S-ROI_mean_ in both groups).*

*(The detailed results were the following: In residual tumor group the mean pretreatment intratumoral ADC was 883 mm2/s for L-ROImean and 580 mm2/s for S-ROImin. The post-EBRT ADC values were 1097 mm2/s (P = 0.006) for L-ROImean and 751 mm2/s (P = 0.017) for S-ROImin. In no residual tumor group the ADC values were before and after EBRT 875 and 1120 mm2/s (P < 0.001) for L-ROImean and 600 and 879 mm2/s (P < 0.001) for S-ROImin respectively. The ADC change was also significant for L-ROImin and S-ROImean in both groups.)*

*To further analyze the prognostic importance of the ADC change in the presence or absence of a post treatment residual tumor, we now made a new variable ‘’Remaining residual tumor after treatments’’ and included this for Cox regression multivariate analyses for overall survival calculations. Larger ADC change rate between pre-treatment and post-EBRT remained still statistically significant, regardless of a tumor residual.*

*Action taken: After including the “Residual tumor” parameter in the Cox regression multivariate analyses for Overall survival, we have checked the results in the manuscript and included the parameters in the supplementary tables. See page 15, lines 292-298 and Supplementary table S2.*

*The following sentence has been added to the discussion, see page 19, lines 378-379: A larger ADC change rate between pre-treatment and post-EBRT was associated with a significantly higher survival rate regardless of tumor residuals.*

In this multivariate analysis, you state that low pre-treatment ADC and high ADC change value were the sole prognostic factors, please add that data not only in the text, but also in Figure, Table. Please also add to in Figure, Table the known prognostic factors for cervical cancer (stage, tumor size, lymph nodes, parametrial invasion, and postoperative adjuvant therapy) which were no significant differences. The results of this multivariate analysis are most important for this study.

*Thank you for the comments. Our Cox regression multivariate analysis results are shown in supplementary tables 2 and 3. S2 table shows overall survival with univariate and multivariate analysis with prognostic factors for cervical cancer (stage, tumor size, lymph nodes, parametria invasion and adjuvant therapy). As described above, we also used the residual tumor variable, but it did not bring any advantage. S3 table shows recurrence free survival at 3 year timepoint. In both univariate and multivariate analysis, the ADC change provided significant prognostic value at that timepoint, but none of the clinical variables remained significant in this analysis.*

*In addition, the supplementary figure S4 illustrates that the known prognostic clinical factors for cervical cancer did not have statistically significant prognostic value in our cohort. This may be due to the small patient population of our study. We submit this new Figure as a supplementary figure. Figure 6 reflects the overall survival and remains as a separate Figure in the manuscript.*

*Supplementary Figure.*  *Univariate analysis of cumulative overall survival according to the different prognostic factors for cervical cancer.*

*A:* *FIGO stage I-IV (P = 0.830):* *blue line indicates stage I, red stage II, green stage III, and orange stage IV.*

*B: Tumor size (P = 0.067): blue line indicates tumor size < 4 cm and red line ≥ 4 cm.*

*C: Lymph nodes (P = 0.239): blue line indicates negative lymph nodes and red line positive lymph nodes.*

*D: Parametria invasion (P = 0.177): blue line indicates parametria invasion, red line no parametria invasion.*

*E: Adjuvant therapy (P = 0.295): blue line indicates that patient received adjuvant therapy, red line indicates no adjuvant therapy.*

*F: Residual tumor (P = 0.257): blue line indicates no residual tumor at 3 months post-treatment MRI and red line indicates residual tumor at 3 month post-treatment MRI*

Discussion:
Of the 28 cases included in the subgroup analysis, the number was reduced to 25 after EBRT. Why did 3 cases drop out? I don't understand why 3 patients dropped out since MRI could have been performed at all 6 points. Please explain.
I understand that 14 cases could not be measured for ADC after EBRT because of small residual tumor, but I do not understand why 3 cases dropped out, assuming that the remaining 11 cases could be measured for ADC.

*Thank you for the question. We are sorry, that we have not been clear in our description of the results. In the subgroup of 28 patients who had MRIs that included DWI and ADC at all six timepoints, 3 of them had no visible tumor after EBRT although DWI and ADC sequences were taken. That is the reason why they dropped out. 25 out of 28 were measured post-EBRT. And later, during brachy therapy when the more excellent effect of the treatment was achieved, more cases dropped out and there was no visible tumor to be measured. Finally, in the 3 month post-treatment imaging only 10 cases had a measurable residual tumor.*

The 14 patients in the group who had ADC measured at all 6 times (residual tumor group) and the 14 patients in the group who had no ADC measured after IGBT (no residual tumor group) should be compared, respectively.
If the rate of increase in ADC after EBRT is significant in both groups, then the rate of increase after EBRT may be able to predict prognosis to some extent, regardless of residual tumor.

*Thank you for this insightful comment. See above our response to the previous comment by the Reviewer. We have the EBRT results from 37patients out of 52. So, we used the whole cohort not only the subgroup of patients. We made a new variable by dividing our cohort into residual tumor groups where there was a residual tumor seen at 3 months post-treatment scan (n = 10) and no residual tumor group (n = 27). We analyzed the ADC change between pre-treatment and post-EBRT scans. There was a significant difference between pre- and post-EBRT ADC values for both groups, regardless of residual tumor.*

*Actions taken: The following sentences are added to the manuscript, page 10, lines 215-222:* *To further analyze the importance of a residual tumor after treatments, the patients with a measurable tumor in the post-EBRT scans (n =37) were divided to those with a residual tumor in the 3-month post-treatment scan (n = 10) and those with no residual tumor in the 3-month post-treatment scan (n = 27). The ADCs were significantly higher at the post-EBRT measurements than in the pretreatment scans in both groups (L-ROI_mean_ P = 0.006, S-ROI_min_ P = 0.017 in residual tumor group and L-ROI_mean_ P < 0.001, S-ROI_min_ P < 0.001 in no residual tumor group respectively. The ADC change was also significant for L-ROI_min_ and S-ROI_mean_ in both groups).*

*We also included this new variable “residual tumor after treatment” in the Cox regression multivariate analysis but the results remained almost same. See supplementary table S2.*

*The following sentence has been added to the discussion, see page 19, line 378-379: A larger ADC change rate between pre-treatment and post-EBRT was associated with a significantly higher survival rate regardless of tumor residuals.*

{Thus, our results could help to schedule the timing of mid-treatment MRI.}
→What does this mean?
Based on the results of this study, I believe that MRI should be performed before the residual tumor disappears for prognostic purposes, and based on the results of multivariate analysis, it should be performed immediately after EBRT.
I think that prognosis cannot be predicted without calculating the ADC change value between pre-treatment and post-EBRT, which was significantly different.

*Thank you for the important comment. That was exactly what we wanted to say. The best time for mid-treatment ADC value measurement is post-EBRT because then most of the tumors are still visible and measurable and the change in ADC is most obvious. During brachy therapy the tumors shrink and even disappear, and there might not be anything to measure and if there is a visible tumor, ADC values do not change so dramatically anymore.*

*Actions taken: The following sentences have been added to Discussion, see page 18, lines 349-352:* *Based on the results of this study, mid-treatment MRI should be performed before the residual tumor disappears for prognostic purposes, and based on the results of multivariate analysis, it should be performed immediately after EBRT. Prognosis can be predicted by calculating the ADC change value between pre-treatment and post-EBRT.*

{The predictive value of the ADC difference before and after EBRT was shown to be statistically significant at the 2 and 3 year timepoints of the survival data} stated.
→Is the cutoff value for this significant difference also 47%?

*Thank you for the important question. The 47 % is the average difference measured in percent between mean pretreatment and mean Post-EBRT ADCs for S-ROI_min._* *The cut-off value for S-ROImin change between pretreatment and post-EBRT ADC that we got from ROC curve using Youden index was 197,5 mm^2^/s. We got the same cut-off value also for 2- and 3-year time points. For L-ROI_mean_ (difference ADC) the cut-off value was 247,5 mm^2^/s.*

*Actions taken: The following sentences have been modified, see page 16, lines 312-317: ADC value difference between the pretreatment and post-EBRT scans was dichotomized using ROC curve to define the Youden index providing the cutoff value of 197.5 mm^2^/s. Blue line indicates group of patients with an increase of over 197.5 mm^2^/s in ADC values (n = 14). Red line indicates group of patients with an increase of less than 197.5 mm^2^/s in ADC* *values (n = 23) An increase in the ADC of over 197.5 mm^2^/s for S-ROI_min_ predicted better overall survival (P = 0.018).*

{Higher pre-treatment ADC values predicted better overall survival at 1 year, but not at 2 or 3 years} stated.
→What is the cutoff value of the pre-treatment ADC value when a significant difference is found? Also, if the cutoff value is lowered, is there likely to be a significant difference at timepoints 2 and 3 years?

*Thank you for the comment. ROC-curve analysis was used to determine the Youden-index, which was used as a cut-off value for dichotomization for each of the timepoints separately. Only pretreatment ADC predicted better overall survival at 1-year timepoint (L-ROI_mean_ cutoff = 791,0 mm^2^/s)(P = 0.05). We repeated the calculations using the 1-year timepoint cutoff (791,0 mm^2^/s) for 2- and 3-year survival analysis, but there were no significant results (2-year timepoint P = 0.113, and 3-year timepoint P = 0.165).*

Conclusion:
{Intratumoral ADCs increase significantly between the pretreatment, post-EBRT, and IGBT MRIs regardless of the ROI delineation method.}
→Only ”pre-treatment and post-EBRT” and “pre-treatment and post-IGBT” were significant differences. Please clarify the description.
Please provide the recommended use of ADC measurement as a prognostic factor based on the results of this study.

*Thank you for the important comment. We clarify our conclusion and want to underly that the use of small ROI is more convenient because it may serve as a prognostic marker for survival.*

*Actions taken: We have added the following sentences to Discussion, page 18, lines 349-352: Based on the results of this study, mid-treatment MRI should be performed before the residual tumor disappears for prognostic purposes, and based on the results of multivariate analysis, it should be performed immediately after EBRT. Prognosis can be predicted by calculating the ADC change value between pre-treatment and post-EBRT.*

*In addition, we have now modified the following sentences: See page 20, lines 416-418: Intratumoral ADCs increase significantly between the pretreatment and post-EBRT as well as between the pretreatment and IGBT MRIs regardless of the ROI delineation method.*

*And page 20, lines 420-422, Conclusion:* *Importantly, in our cohort, the greater increase in ADC values between pretreatment and post-EBRT values measured using small standard-size ROIs, seemed to provide a surrogate marker for better overall and recurrence free survival.*
